# Hypoxemia and Right-to-Left-Shunt in Patient with Antiphospholipid Syndrome: A Case Report with Multimodality Imaging Findings and Literature Review

**DOI:** 10.1155/2016/2092084

**Published:** 2016-02-29

**Authors:** Mnahi Bin Saeedan, Mashael Alrujaib, Ahmed L. Fathala

**Affiliations:** King Faisal Specialist Hospital and Research Center, Department of Radiology, P.O. Box 3354, Riyadh 11211, Saudi Arabia

## Abstract

This is a case report of an extremely rare cause of superior vena cava syndrome with systemic-to-pulmonary venous shunts, illustrated using different imaging modalities with successful SVC and IVC dilatation and stenting.

## 1. Introduction

Superior vena cava (SVC) obstruction might be caused by different etiologies. Among these, lung cancer and lymphoma are the most frequent causes. Other benign processes such as central venous catheter and fibrosing mediastinitis can cause SVC obstruction. Collateral venous channels are usually formed to restore venous return [1–4]. Systemic-to-pulmonary venous shunts (SPVS) might be developed in case of severe and long-standing SVC obstruction [5–9].

Here, we present a rare cause of SPVS in patient with antiphospholipid syndrome, Budd-Chiari Syndrome, and SVC obstruction which was successfully treated with SVC and IVC dilatation and stenting. We illustrated the SVC obstruction and SPVS using different imaging modalities.

## 2. Case Report

A 22-year-old female presented to the hospital with a history of progressive dyspnea, fatigue, and chest and neck tightness. She had a history of antiphospholipid syndrome, Budd-Chiari Syndrome, and end stage renal failure. On physical examination, the patient was cyanotic with a heart rate of 125 beats/min, respiratory rate of 22/min, blood pressure of 82/50 mmHg, and oxygen saturation of 86% on room air and 95% on 15 liters nonrebreather oxygen mask.

**Figure 1:**
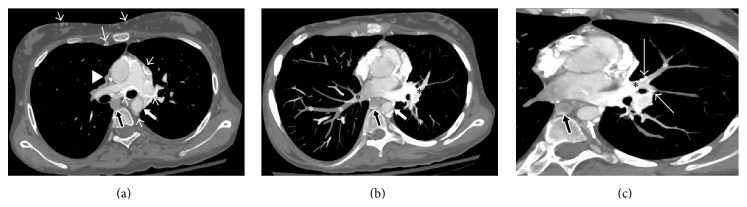
Enhanced axial chest CT image (a) and oblique axial thin-slab maximum-intensity-projection (MIP) images (b, c) inferior to (a) show thrombosed and stenosed SVC (arrowhead) and distended and occluded azygos vein (black block arrow). There are chest wall, accessory hemiazygos, and paravertebral, intense mediastinal venous collaterals (short arrows). Central superior pulmonary veins (black asterisks) are opacified through adjacent collateral (long white arrows) as compared to nonopacified peripheral portions (white asterisks). Note the contrast filled aorta (white block arrow).

Chest computed tomography (CT) angiography was performed to rule out pulmonary emboli. The exam was performed using IV iodinated contrast (Xenetix) via right antecubital vein with a power injector. Images were acquired in helical mode using multidetector CT scan (GE discovery, CT 750 HD) with slice thickness of 0.625 mm. Axial, coronal, and sagittal reconstructions and multiplanar reformats (MPR) were performed. The exam showed complete nonopacification of the SVC and left brachiocephalic vein. The upper azygos vein was distended and not opacified as compared to the opacified inferior part. There were extensive collaterals draining into the right atrium through coronary sinus and some appeared to drain directly into the right atrium. Furthermore, the exam showed numerous strongly enhancing multiple hilar, mediastinal, and pericardial venous plexus around the bronchi, pulmonary arteries, and pulmonary veins with subtle early filling of the central pulmonary veins as compared to peripheral segments leading to the left heart chambers and aorta with early opacification (Figure 1). There was no filling defect to suggest pulmonary embolism. The exam revealed multiple collateral venous pathways, such as the azygos and hemiazygos veins, internal and lateral thoracic veins, and vertebral venous plexus.

**Figure 2:**
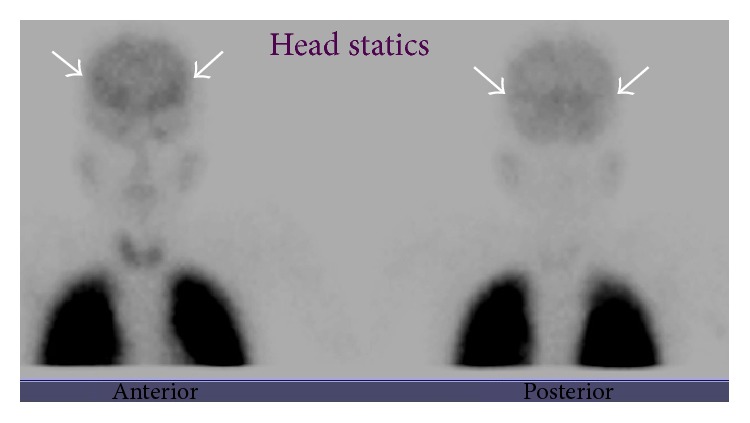
Anterior and posterior static head images of lung perfusion scan images with Tc-99m macroaggregated albumin (MAA) show focal tracer uptake noted at the region of brain (white arrows) and stomach and faint renal uptake (not shown) indicative of right-to-left shunt.

Echocardiography with limited views was performed with saline injection via the right antecubital vein and revealed echo bubble in both sides of the heart, more on the left side, suggestive of venous connection to left atrium. Lung perfusion scan with Tc-99m MAA was performed and showed focal tracer uptake at the region of the brain, stomach, and both kidneys indicating right-to-left shunt (Figure 2). Cardiac magnetic resonance imaging (MRI) was requested to assess and quantify the shunt. There was a sluggish flow of the pulmonary artery branches in the phase contrast necessitating reduction in the velocity encoded gradient echo from 150 cm/sec (routinely used for arteries) to 100 cm/sec (routinely used for veins). The average velocities of the MPA, RPA, and LPA were reduced and measured 5.45 cm/sec, 6.37 cm/sec, and 7.76 cm/sec, respectively. The net forward volume to body surface area was 7 mL/m^2^ for the MPA and 15 mL/m^2^ for the ascending aorta with a Qp/Qs ratio of 0.5. An attempt to assess the pulmonary veins was initiated; however, the patient was unable to complete the exam.

Conventional venogram was performed by the cardiologist and confirmed SVC complete obstruction at the SVC/RA junction and narrowing of the IVC with the narrowest diameter measuring 6.6 mm. The patient then underwent successful SVC and IVC dilatation and stenting. The patient was extubated immediately following the procedure and was clinically stable with normal oxygen saturation on room air and complete resolution of her cyanosis.

## 3. Discussion

SVC obstruction associated with SPVS is usually caused by primary or secondary malignant processes [1, 7, 8]. However, it has been reported with other rare benign entities [5, 6, 9].

We present a case of antiphospholipid syndrome causing BCS with IVC and SVC thrombosis with progressive shortness of breath and hypoxemia due to SVC syndrome and SPVS. Upon extensive literature review, we found that this is the fourth reported case of BCS associated with SVC obstruction [5, 6, 10]. Two of these cases were associated with SPVS [5, 6] and one was known to have antiphospholipid syndrome similar to our case [6].

There are several factors which make this case interesting. First of all, it is an extremely rare benign cause of SVC syndrome and SPVS. Secondly, multiple findings are illustrated in several imaging modalities including enhanced CT scan, lung perfusion scan, and cardiac MRI. The third interesting point is that this patient had a successful IVC and SVC dilatation and stenting with significant clinical improvement similar to one previous reported case [6]. The fourth point is that this case shows predominately central multiple mediastinal venous plexus collaterals around the bronchi, pulmonary arteries, and pulmonary veins resulting in early filling of central pulmonary veins with paucity of contrast in peripheral pulmonary veins unlike the other two reported cases [5, 6]. Combinations of these diagnostic and therapeutic findings have not been reported in the previous two cases.

SPVS imaging findings reported in several modalities include radionuclide lung perfusion scan using 99m-Tc-macroaggregated albumin [11], conventional venography [12], and CT scan [2, 3]. Nevertheless, CT venography has been widely used as it may reveal the cause of SVC syndrome, determine the exact level of venous occlusion, and depict the collateral pathways [1–4].

In SVC syndrome, the head, neck, and upper limbs venous drainage bypasses the thrombosed SVC by the azygos, the lateral thoracic, internal mammary, and vertebral venous pathways. Unusual collaterals established between systemic and pulmonary veins presumably due to increased venous pressures causing a right-to-left shunt [1, 3]. SPVS may cause hypoxemia, systemic embolism through systemic-to-pulmonary venous connections or brain abscess. These unusual collaterals with their anatomical substrates have been discussed in the literature of SVC syndrome [1–4]. These shunts may be further classified as congenital, anatomic, or acquired. In this case, no anomalous venous return was identified. Anatomically, there are venous connections between bronchial and pulmonary veins through preexisting bronchial venous plexuses and between the azygos-hemiazygos system and the pleurohilar bronchial veins through the intervening valves. Increase in systemic venous pressure, as SVC obstruction, may result in reversal of flow and a right-to-left shunt. This anatomic cause is the most likely explanation of these patients' predominantly central SPVS. However, another theory may explain the predominant peripheral and pleural-based SPVS with possible pleural thickening that has been seen in other reported cases. It is proposed that it is inflammatory in nature, causing newly formed vessels bridging subpleural pulmonary veins and intercostal veins through pleural adhesions [1].

## 4. Conclusion

In patients with antiphospholipid syndrome related BCS, bicaval obstruction may lead to SPVS formation. SPVS might cause hypoxemia or be complicated by systemic embolism or brain abscess. Radiologist and referring physician should be familiar with such appearance and aware of such potential complications. Stenting the SVC and the IVC stenotic zones may provide significant clinical improvement.
